# Insights into T‐cell dysfunction in Alzheimer's disease

**DOI:** 10.1111/acel.13511

**Published:** 2021-11-01

**Authors:** Linbin Dai, Yong Shen

**Affiliations:** ^1^ Institute on Aging and Brain Disorders The First Affiliated Hospital of USTC Division of Life Sciences and Medicine University of Sciences and Technology of China Hefei China; ^2^ Neurodegenerative Disease Research Center University of Science and Technology of China Hefei China; ^3^ Hefei National Laboratory for Physical Sciences at the Microscale University of Science and Technology of China Hefei China

**Keywords:** Alzheimer's disease, hallmarks, neuroinflammation, risk factors, T cells

## Abstract

T cells, the critical immune cells of the adaptive immune system, are often dysfunctional in Alzheimer's disease (AD) and are involved in AD pathology. Reports highlight neuroinflammation as a crucial modulator of AD pathogenesis, and aberrant T cells indirectly contribute to neuroinflammation by secreting proinflammatory mediators via direct crosstalk with glial cells infiltrating the brain. However, the mechanisms underlying T‐cell abnormalities in AD appear multifactorial. Risk factors for AD and pathological hallmarks of AD have been tightly linked with immune responses, implying the potential regulatory effects of these factors on T cells. In this review, we discuss how the risk factors for AD, particularly Apolipoprotein E (ApoE), Aβ, α‐secretase, β‐secretase, γ‐secretase, Tau, and neuroinflammation, modulate T‐cell activation and the association between T cells and pathological AD hallmarks. Understanding these associations is critical to provide a comprehensive view of appropriate therapeutic strategies for AD.

## Graphical abstract

T‐cell dysfunction is associated with Alzheimer's disease pathology and takes several forms and involves many different factors. In this review, we highlight that T‐cell dysfunction leads to neuroinflammation, a critical modulator of AD pathology. We review the literature pertaining to AD risk factors which contribute to T‐cell dysfunction, including Apolipoprotein E (ApoE), Aβ, α‐secretase, β‐secretase, γ‐secretase, Tau, and neuroinflammation, with a focus on understanding the role of T‐cell dysfunction in AD progression and the possibility of T cells being a target for successful AD treatments

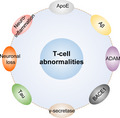

## 1 INTRODUCTION

Alzheimer's disease (AD), which usually progresses to dementia, is the most prevalent neurodegenerative disease in older adults (Hardy & Higgins, 1992). Symptoms of the disease manifest mainly as deficits in cognitive function, including memory loss, impairment of language, misidentifications, and behavioral disturbances (Burns et al., 2002). Neuropathologically, AD is remarkably characterized by two proteinaceous aggregate hallmarks, including tau‐hyperphosphorylation‐induced intracellular neurofibrillary tangles (NFTs) and extracellular depositions of amyloid plaques induced by beta‐amyloid peptide (Aβ) (Ittner & Gotz, 2011), which both contribute to synaptic damage and neuronal loss.

In the past few decades, various groups have devoted enormous efforts to explore AD pathogenesis and find key risk factors for the prevention and treatment of AD. However, it is disappointing that almost all AD‐related clinical trials to date have failed to reverse cognitive decline and/or brain pathology. Undoubtedly, it is gratifying that there are different reasonable evidence‐based hypotheses relating to underlying causes, such as the amyloid cascade hypothesis, the tau hypothesis, the Apolipoprotein E (ApoE) hypothesis, and the neuroinflammation hypothesis (Jiang et al., 2014; C. C. Liu et al., 2013; Lue et al., 2001; Morales et al., 2014; L. B. Yang et al., 2000).

**FIGURE 1:**
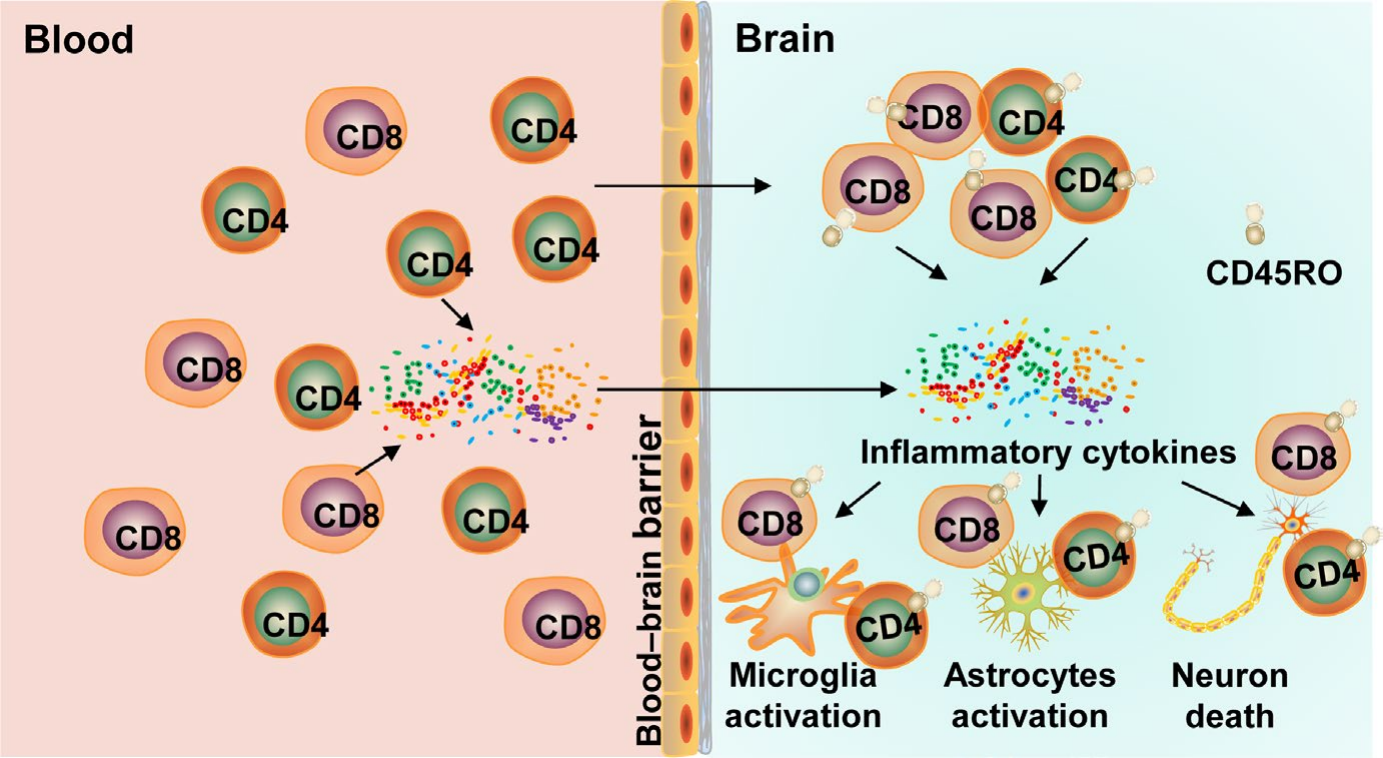
Summary of T‐cell roles in AD. With the development of AD, T‐cell infiltration into the brain increases, and a large number of inflammatory cytokines derived from T cells in the peripheral blood also enter the brain, which eventually exacerbate neuroinflammation and accelerate neuronal death

Although it is widely accepted that neuroinflammation in AD is mediated by microglia and astrocytes, mounting evidence shows that T cells are involved in regulating the inflammatory response in AD through, but not limited to, the following two aspects (Figure 1). First, during AD progression, the permeability of the blood–brain barrier (BBB) gradually increases due to decreased expression of the tight junction molecules ZO1 and occludin in vascular endothelial cells (Carrano et al., 2011; Cheng et al., 2014; Marco & Skaper, 2006). In addition, there is elevated peripheral T‐cell expression of chemokine receptors, such as C‐C motif chemokine receptor type 2 (CCR2), C‐C motif chemokine receptor type 5 (CCR5), and C‐X‐C motif chemokine receptor 2 (CXCR2) (Goldeck et al., 2013; Liu et al., 2010; Town et al., 2005a). Both of these abnormal changes promote T‐cell penetration into brain parenchyma. More important, activated CD8+ T and CD4+ T cells, the two major T‐cell subsets, are neurotoxic and can induce substantial neuronal death through mechanisms dependent on cell–cell contact involving Fas ligand (FasL), lymphocyte function‐associated antigen‐1 (LFA‐1), and CD40 (Giuliani et al., 2003). Increased T‐cell infiltration also promotes crosstalk between T cells and microglia in a process dependent on major histocompatibility complex (MHC) class II, leading to further acceleration of neuroinflammation (Schetters et al., 2017). T‐cell‐derived cytokines can also impact local astrocyte‐expressed chemokine function in inflammatory and neurodegenerative diseases (Williams et al., 2020). Second, peripheral T cells can modulate glial cell neuroinflammation mediated by the release of proinflammatory factors into the central nervous system (CNS), such as interferon gamma (IFN‐γ) (Town et al., 2005b). Activation of peripheral T cells in AD patients is elevated compared to healthy controls, and these T cells further promote peripheral blood mononuclear cells (PBMCs) to release proinflammatory factors including IL‐6, tumor necrosis factor α (TNFα), and IL‐1, all of which are essential immune modulators in AD pathology (Mietelska‐Porowska & Wojda, 2017; Tan et al., 2002).

Despite accumulating evidence suggesting that T cells participate in immunological and pathological stages of AD concomitant with changes in T‐cell phenotype, the mechanism of T‐cell abnormality in AD remains unknown. In this review, we summarize T‐cell dysfunction in AD and demonstrate the association between T cells and the critical pathological features of AD. Furthermore, we propose that aberrant activation of T cells contributes to AD pathogenesis, and that the critical elements of AD can also mediate biological processes involving T cells.

## 2 T‐CELL ABNORMALITIES IN AD

Although the mechanisms by which T cells contribute to AD pathophysiology are unclear, considerable work has shown that normal T‐cell function and T‐cell markers in both AD mouse models (Table 1) and AD patients are different when compared to measures from respective control groups (Table 2). As early as 1981, for example, Miller and colleagues first demonstrated that concanavalin A (ConA)‐induced T‐cell suppression in AD patients was significantly elevated and lymphocyte proliferation was lower when compared with an elderly control group (Miller et al., 1981). However, another group found no difference in phytohaemagglutinin (PHA)‐induced T‐cell proliferation between T cells derived from AD patients and those from age‐matched controls (Leffell et al., 1985). This discrepancy may be accounted for by different types or intensities of mitogen stimulation used. Although interpretation of mitogen‐induced T‐cell activation is debated due to such discrepancies, variation in T‐cell populations in peripheral blood from both AD mouse models and AD patients appears convincing, as described below. A higher ratio of CD4+/CD8+ T cells was found in the in peripheral blood of AD patients and AD mouse models compared to relevant controls, concomitant with lower CD8+ T‐cell counts and lower total CD3 expression (Hu et al., 1995; Pirttila et al., 1992; Schindowski et al., 2007; Shalit et al., 1995; St‐Amour et al., 2014, 2019). Furthermore, analyses of peripheral blood from AD patients have shown that the number of IL‐2R+, HLA‐DR+, CD25+, and CD28+T cells was significantly higher than controls (Ikeda et al., 1991; Lombardi et al., 1999; Speciale et al., 2007), indicating an immune response in the peripheral system. Similarly, a consistent finding is that the numbers of CD4+ and CD8+ T cells in the brain parenchyma and cerebrospinal fluid (CSF) of AD patients are significantly higher than normal, with CD8+ T cells having the advantage over CD4 + T cells in absolute numbers, and that both subtypes exhibit the CD45RA‐CD45RO+ phenotype (Ferretti et al., 2016; Laurent et al., 2017; Merlini et al., 2018; Rogers et al., 1988; Togo et al., 2002), indicating that infiltrating T cells were activated and may be cytotoxic.

**TABLE 1:** Summary of T‐cell abnormalities in AD animal models (studies in chronological order)

Study	Mouse model	Findings (AD vs WT)
(Schindowski et al., 2007)	Thy1‐APP751SL · HMG‐PS1M146L mouse	Increased CD4/CD8‐ratio in PBMC
		Decreased CD3 and CD8 surface expression in PBMC
		Decreased mitogen‐induced activation and proliferation in CD8+ T cells
(Browne et al., 2013)	APPxPS1 mouse	Increased Th1 and Th17 subsets infiltrate the brain
(Robinson et al., 2013)	APPxPS1 mouse	Increased oxidative stress in T cells with age
(Zhang et al., 2013)	Injected Aβ1‐42 rat	Increased Th17 subsets infiltrate the brain
		Increased expression of IL−17, IL−22, and FasL by Th17 cells
(St‐Amour et al., 2014)	3xTg‐AD mouse	Decreased CD4+ and CD8+ T cells in the blood
		Increased CD4/CD8‐ratio in blood
(McManus et al., 2014)	APPxPS1 mouse	Increased IFNg+and IL−17+T cells infiltration into brains after a respiratory infection
(Baruch et al., 2015)	5xFAD mouse	Increased Treg cells along disease progression in in spleen
(Baek et al., 2016)	3xTg‐AD mouse	Increased CD4/CD8‐ratio in in spleen
(Ferretti et al., 2016)	Tg2576 mouse	Increased CD3+T cells infiltrate the brain
	APPxPS1 mouse	Increased CD3+T cells infiltrate the brain
	ArcAb mouse	Increased CD3+T cells infiltrate the brain (CD8+ T being the predominant)
		Decreased percentage of IFN+cells in the CD4+ and CD8+ T cells in brain
(Laurent et al., 2017)	THY‐Tau22 mouse	Increased CD8+ T cells infiltration into the parenchyma
(Saksida et al., 2018)	5xFAD mouse	Increased CD4+ T cells in Peyer's patches (PP) and mesenteric lymph node (MLN)
		Decreased capacity of T cells to produce IL−17
(St‐Amour et al., 2019)	3xTg‐AD mouse	Decreased T cells numbers in blood
		Increased activation along with Th17 polarization of T cells
(Wang et al., 2019)	5xFAD mouse	Increased T cells, particularly Th1 cells infiltrate the brain
		Increased the differentiation and proliferation of Th1 cells
(Yang et al., 2020)	APP/PS1 mouse	Increased proportions of CD4+CD25+Foxp3+ Tregs in the spleen
(Unger et al., 2020)	APP/PS1 mouse	Increased numbers of CD8+ T cells in brain parenchyma
(Sanchez et al., 2020)	PS19/5xFAD mouse	Increased numbers of memory CD8+ T cells in brains

**TABLE 2:** Summary of T‐cell abnormalities in AD patients (studies in chronological order)

Study	Population	Findings (AD vs other controls)
(Miller et al., 1981)	24 AD	Decreased mitogen‐induced T cells proliferation
	32 age‐matched controls	Increased Con A‐induced T cells suppression responses
(MacDonald et al., 1982)	41 AD	Decreased UCHT3 +T‐cell number in the blood
	41 age‐matched controls	
	41 younger controls	
(Skias et al., 1985)	16 AD	Decreased CD8+ T cell‐mediated suppressor function using a PWM‐induced IgG secretion assay
	14 age‐matched controls	
(Leffell et al., 1985)	30 AD	No changes in PHA‐induced T cells proliferation compared to age‐matched controls
	30 age‐matched controls	No changes in T‐cell subsets compared to age‐matched controls
	20 younger controls	Decreased responsiveness of T cells from AD and 30 age‐matched controls compared to younger controls
(Torack 1986)	95 AD‐DTH treated	Increased suppressor cells activity
	61 control‐DTH treated	Decreased Con A‐induced T‐cell responses
	19 AD‐Con A treated	
	11 control‐ Con A treated	
(Gibson et al., 1987)	9 AD	Decreased mitogen‐induced calcium uptake by T cells
	9 age‐matched controls	
(Bartha et al., 1987)	15 AD	Decreased the AChE activity of T cells in the blood
	40 multi‐infarct dementia	
	8 alcoholic dementia	
	30 age‐matched controls	
(McGeer et al., 1988)	10 AD	Increased the presence of CD4+ and CD8+ T cells in capillaries of the brain (CD8+ T much more prevalent then CD4+ T)
	5 age‐matched controls	
(Rogers et al., 1988)	10 AD	Increased the presence of CD4+ and CD8+ T cells in the brain parenchyma and blood vessels
	6 age‐matched controls	
(Leonardi et al., 1989)	26 AD	Increased T‐cell proliferative response in AMLR
	10 age‐matched controls	
(Adunsky et al., 1991)	22 AD	Increased cytosolic calcium in resting and activated T cells
	6 multi‐infarct dementia	
	19 age‐matched controls	
(Rocca et al., 1991)	20 early‐onset AD	Decreased T cells 3H‐NMS binding sites
	15 late‐onset AD	
	86 other neurological disorders	
	60 age‐matched controls	
(Ikeda et al., 1991a)	13 AD 13 age‐matched controls	Increased the ratio of T‐cell subsets of CD4+IL−2R+, CD4+HLA‐DR+, CD8+HLA‐DR+, CD8+HLA‐DR+in peripheral blood
		Decreased the ratio of T‐cell subsets of CD4+CD45R+ in peripheral blood
(Ikeda et al., 1991b)	13 AD 13 age‐matched controls	Increased the ratio of T‐cell subsets of CD4+CD45R‐, CD4+HLA‐DR+in peripheral blood
(Pirttila et al., 1992)	31 AD	Decreased CD8+ T cells number in peripheral blood
	35 age‐matched controls	Increased the ratio of CD4+/CD8+ T cells in peripheral blood
	136 other neurological disorders	
(Grossmann et al., 1993)	6 familial AD	Decreased intracellular calcium response in CD4+ T cells
	39 AD	
	11 DS	
(Hartmann et al., 1994)	14 AD	Increased baseline cytosolic calcium in T cells
	14 age‐matched controls	
	14 younger controls	
(Nijhuis et al., 1994)	30 AD	Decreased sensitivity to DEX in T cells
	30 age‐matched controls	
(Adunsky et al., 1995)	30 AD	Increased cytosolic calcium in resting and activated PBMC
	27 age‐matched controls	
(Eckert et al., 1995)	24 AD	Decreased the amplifying effect of Aβ25‐35 on calcium signaling in PBMC
	20 age‐matched controls	
	16 younger controls	
(Hu et al., 1995)	20 AD	Decreased CD8+ T cells of PBMC in AD and in other forms of dementia
	17 other dementia	Increased the ratio of CD4+/CD8+ T cells in peripheral blood
	23 age‐matched controls	
	17 younger controls	
(Shalit et al., 1995)	12 Mild AD	Increased the ratio of CD4+ T cell of PBMC in moderately AD compared with age‐matched controls
	13 Moderately AD	Increased the ratio of HLA‐DR+cell of PBMC in moderately AD compared with age‐matched controls
	13 age‐matched controls	Decreased lymphocytes proliferation but increased IL−2 synthesis induced by PHA in moderately AD compared with age‐matched controls
(Kell et al., 1996)	24 AD	The percent of IgM+T cells was negatively correlated with MMSE scores
	9 other dementia	
	20 age‐matched controls	
(Singh 1996)	11 AD	Increase sICAM−1 and sCD8 in AD plasma
	10 age‐matched controls	
(Bongioanni et al., 1997)a	35 AD	Decreased number of T‐cell IFN‐γ receptors in PBMC
(Bongioanni et al., 1997)b	35 age‐matched controls	
	35 AD	Increased number of T‐cell TNFα receptors(both TNFR1 and TNFR2) in PBMC
	35 age‐matched controls	Increased number of T‐cell TNFα receptors(both TNFR1 and TNFR2) in PBMC Increased T‐cell IL‐ 6 receptor binding
(Bongioanni et al., 1998)c	35 AD	
	35 age‐matched controls	
(Lombardi et al., 1999)	45 AD	Decreased CD8+ T cell in PBMC
	45 age‐matched controls	Increased CD4, CD25, and CD28 antigen expression in PBMC
		Increased Fas antigen (CD95) expression on CD4+ T cell by anti‐CD3 and hyperthermia mediated‐apoptosis
		Decreased Fas antigen (CD95) expression on CD8+ T cell by anti‐CD3 and hyperthermia mediated‐apoptosis
(Sulger et al., 1999)	27 AD	Increased calcium responses of T cells induced by PHA
	27 age‐matched controls	
	27 younger controls	
(Eckert et al., 2001)	18 AD	Increased apoptotic nucleosomes in native lymphocytes
	14 age‐matched controls	Increased apoptotic nucleosomes in activated lymphocytes
(Stieler et al., 2001)	18 AD	Decreased T‐cell proliferative response induced by PHA
	45 age‐matched controls	
(Tan, et al., 2002)	46 AD	Decreased CD45RA expression and increased CD45RO/RA ratio in CD4+ T cells
	37 cognitively abnormal	
	90 age‐matched controls	
(Togo et al., 2002)	21 AD	Increased number of T cells(CD45RA‐CD45RO+,activeted)entering the brain
	36 other dementia	
	3 age‐matched controls	
(Giubilei et al., 2003)	30 AD	Decreased T cells responses induced by microbial peptides and human mitochondrial
	30 age‐matched controls	Decreased T cells responses induced by microbial peptides and human mitochondrial Increased T cells responses to Aβ with age
(Monsonego et al., 2003)	29 AD	Decreased T cells responses induced by microbial peptides and human mitochondrial Increased T cells responses to Aβ with age Increased frequencies of Aβ‐specific CD4+ T cells in PBMC
	22 age‐matched controls	
	13 younger controls	
(Panossian et al., 2003)	15 AD	Decreased telomere length of T cells
	15 age‐matched controls	Increased lymphocytes proliferation induced by PHA
(Lombardi et al., 2004)	88 AD	Increased DNA fragmentation of T‐cell exposure to IgM anti‐Fas
(Lombardi et al., 2004) (Dorszewska et al., 2005)	24 age‐matched controls	Increased expression of Fas mRNA and surface Fas receptor on CD45RO+ T lymphocytes
(Lombardi et al., 2004) (Dorszewska et al., 2005)	34 AD	Increased expression of p53, Bax, PARP in PBMC
	44 age‐matched controls	Decreased expression of Bcl−2 in PBMC
(Iarlori et al., 2005)	40 AD	Decreased expression and production of MCP−1 in PBMC
	20 age‐matched controls	Increased expression and production of RANTES in PBMC
(Schindowski et al., 2006)	34 AD	Increased apoptosis in CD4+ T cells
	34 age‐matched controls	Increased expression of Bcl2 in T cells in mild AD
(Zana et al., 2006)	22 AD	Decreased apoptosis in CD4+ T cells induced by UVB
	12 age‐matched controls	
(Man et al., 2007)	83 AD	Increased expression of MCP−1 in peripheral T cells
	70 age‐matched controls	Increased T cells transmigrating the HBMEC monolayer
(Schindowski et al., 2007)	34 AD	Increased CD4/CD8‐ratio in PBMC
	34 age‐matched controls	Decreased CD8 and CD3 expression in PBMC
		Increased T‐cell tyrosine phosphorylation induced by mitogen
		Increased number of CD45RO+ CD8 + T cells
		Increased T cells reactivity in PBMC
(Speciale et al., 2007)	29 mild AD	Decreased CD8+CD28‐ cells in PBMC
	22 moderately AD	Increased CD8+CD28+ cells and CD8+CD71+ cells in PBMC
	51 age‐matched controls	Decreased IL−10 production by PBMC after Aβ stimulate
(Ciccocioppo et al., 2008)	40 AD	Increased phosphorylation of protein kinase C in Aβ activated T cells
	20 age‐matched controls	
	20 younger controls	
(Larbi et al., 2009)	12 AD	Decreased naïve T cell in PBMC
	6 age‐matched controls	Increased EM and TEMRA T cells
	20 younger controls	
(Miscia et al., 2009)	20 early AD	Increased T‐cell reactivity to Aβ1‐42
	20 severe AD	Increased phosphorylation of PKC‐δ and PKC‐ζ in Aβ activated T cells
	20 age‐matched controls	
	20 younger controls	
(Liu et al., 2010)	58 AD	Increased CXCR2 expression on peripheral T cells
	47 age‐matched controls	
(Pellicano et al., 2010)	40 AD	Increased CD69 expression on Aβ activated T cells
	25 age‐matched controls	Increased CCR2 and CCR5 expression on Aβ activated T cells
(Saresella et al., 2010)	25 AD	Increased total Treg and PD1+Treg cells
	25 MCI	
	55 age‐matched controls	
(Saresella et al., 2011)	38 AD	Increased activity of Th17 and Th9 cells after Aβ stimulate
	34 MCI	
	40 age‐matched controls	
(Goldeck et al., 2013)	23 AD	Increased CCR4, CCR5, and CCR6 expression on peripheral T cells
	20 age‐matched controls	Increased the shift of early‐ to late‐differentiated CD4+ T cells
(Salani et al., 2013)	13 AD	Increased IL−18Rβ expression on peripheral T cells
	24 MCI	
	25 age‐matched controls	
(Westman et al., 2013)	50 AD	Decreased proportion of CMV Specific CD8+ T cells
	50 age‐matched controls	
(Westman et al., 2014)	30 AD	Increased PBMC inflammatory response in CMV seropositive patients
	35 age‐matched controls	
(Busse et al., 2015)	24 AD	Decreased VGF expression on peripheral T cells
	14 age‐matched controls	
(Le Page et al., 2017)	15 AD	Decreased proportion of Treg cell compare with MCI
	13 MCI	
	13 age‐matched controls	
(Terzioğlu et al., 2017)	30 early‐onset AD	Increased mitochondrial depletion in peripheral CD4+ T cells
	30 late‐onset AD	
	30 age‐matched controls	
(Liu et al., 2018)	17 AD	Increased Let−7b expression on CSF T cells
	36 MCI	Increased proportion of T cells in CSF
	41 age‐matched controls	
(Merlini et al., 2018)	9 AD	Increased extravascular T cells (mostly of the CD8+) in the brain
	10 age‐matched controls	
(Oberstein et al., 2018)	14 AD	Increased proportion of Th17 cells in PBMC
	27 MCI	
	13 age‐matched controls	
(Rakic et al., 2018)	40 AD+systemic infection	Decreased T cells recruitment to the brain after encounter systemic infection
	28 AD‐ systemic infection	
	16 controls+systemic infection	
	24 controls‐ systemic infection	
(Tramutola et al., 2018)	19 AD	Increased protein nitration profile of T cells
	19 age‐matched controls	
(Ciccocioppo et al., 2019)	10 AD	Decreased total and resting Tregs in PBMC
	8 AD age‐matched controls	
	10 MS	
	8 MS age‐matched controls	
(Wang et al., 2019)	31 MCI due to AD	Increased Th1 cell frequency in the blood
	40 age‐matched controls	
(Gate et al., 2020)	28 AD	Increased numbers of CD8+ TEMRA cells in PBMC
	8 PD	Increased clonally expanded CD8+ TEMRA cells in the CSF
	31 MCI	
	97 age‐matched controls	
(D’Angelo et al., 2020)	11 AD	Increased CCR6+ and CCR4+ CD4+ T cells in the blood
	6 VaD	
	6 mix dementia	
	17 age‐matched controls	
(Faridar et al., 2020)	46 AD	Decreased suppressive function of regulatory T cells from peripheral blood
	42 MCI	Decreased CD25 mean fluorescence intensity in regulatory T‐cell population
	41 age‐matched controls	
(Amin et al., 2020)	31 AD	Increased numbers of Th cells in PBMC
	31 DLB	
	31 age‐matched controls	
(Dhanwani et al., 2020)	51 AD	No difference of T‐cell responses to neural autoantigens
	54 age‐matched controls	

Abbreviations

AD: Alzheimer's Disease; Con A: Concanavalin A; PWM: Pokeweed mitogen; IgG: Immunoglobulin G; PHA: Polyhydroxyalkanoates; DTH: Delayed type hypersensitivity; AChE: Acetylcholinesterase; AMLR: Autologous mixed lymphocyte reaction; 3H‐NMS: 3H ‐N‐methyl‐scopolamine; DS: Down's syndrome; DEX: dexamethasone; PBMC: Peripheral blood mononuclear cells; sICAM‐1: soluble intercellular adhesion molecule‐1;sCD8: soluble CD8; TNFα: Tumor necrosis factor; TNFR1: Tumor necrosis factor receptor1; TNFR2: Tumor necrosis factor receptor2; MMSE: Mini Mental State Examination; MCP‐1: Monocyte chemoattractant protein‐1; RANTES: Chemokine (C‐C motif) ligand 5 (CCL5); UVB: ultraviolet B; HBMEC: Human brain microvascular endothelial cells; EM: Effector memory; TEMRA: Terminally differentiated effector memory; PKC:Protein kinase C; MCI: mild cognitive impairment; CMV: Cytomegalovirus; CSF: Cerebrospinal fluid; MS: Multiple Sclerosis. VaD: Vascular dementia. DLB: Dementia with Lewy bodies.

In addition to apparent changes in the number and proportion of AD‐derived T cells, alterations have also been observed in the intracellular signaling pathway in T cells from AD patients, such as changes in calcium response. For example, there are in vitro data which show that both baseline cytosolic calcium and PHA‐induced calcium responses in T cells from AD patients were higher than measures from control groups (Adunsky et al., 1995; Sulger et al., 1999). However, beta‐amyloid fragment Aβ25‐35 led to a substantial reduction in mitogen‐induced calcium signaling rise in the PBMC of AD patients compared with that of age‐matched controls (Eckert et al., 1995). Therefore, the precise mechanism of calcium homeostasis in T cells may depend on the specific type of stimulus and the local microenvironment but may not rely only on AD status.

T cells from AD patients and AD mouse models are hyperactive to Aβ stimulation, which increases expression of activation marker CD69 and enhances cytokine production (Ciccocioppo et al., 2008; Miscia et al., 2009; Pellicano et al., 2010). Cytokines secreted by activated T cells are significant modulators of microglia and astrocyte function in AD. For example, IL‐10, TNF‐α, IL‐6, IL‐1β, and monocyte chemoattractant protein‐1 (MCP‐1) released from T cells are markedly elevated in AD patients (Lombardi et al., 1999; Man et al., 2007), and microglia and astrocytes are the main targets of these inflammatory factors (Hanisch, 2002; Ramesh et al., 2013). Thus, dysregulation of T cells which overexpress these cytokines is potentially harmful and likely contributes to chronic neuropathology in AD.

Furthermore, proinflammatory cytokines may be responsible for the prevalence of elevated Th1 and Th17 cells in AD (Browne et al., 2013; Oberstein et al., 2018; Saresella et al., 2011; Zhang et al., 2013). Th1 and Th17 cells are two major proinflammatory T‐cell subtypes which are typically elevated in neurodegenerative diseases, including AD. In addition, Th1 cells are significant sources of IFN‐γ secretion. At the same time, microglia and astrocytes can be activated by IFN‐γ and disturb cell homeostasis, thereby contributing to Aβ deposition, impaired synaptic plasticity, and acceleration of cognitive deficits in APP/PS1 mice (Browne et al., 2013; R. J. Kelly et al., 2013). However, injection of Aβ‐specific Th1 cells into 5xFAD mice enhances Aβ uptake due to T‐cell‐activation‐induced expansion of brain‐endogenous MHCII+microglia, which exhibit stronger phagocytic activity (Mittal et al., 2019). These findings suggest that the function of Th1 cells in AD may be linked to disease stage. Therefore, manipulation of Th1 cells at an appropriate period may be a helpful approach for AD therapy.

Alternatively, elevated Th17 cells in AD may also be detrimental and induce neuronal apoptosis (Zhang et al., 2013), through the release of proinflammatory factors such as FasL, IL‐17, and IL‐22. In contrast to the destructive function of Th17 cells, the adoptive transplantation of Th2 cells (one of the immunosuppressive T‐cell subtypes) into APP/PS1 AD mice benefits cognitive function and reduces pathological features (Cao et al., 2009). The function of regulatory T cells (Tregs, another immunosuppressive T‐cell subtype) has also been described in AD. Transient depletion of Tregs in 5xFAD mice is beneficial for Aβ clearance and cognitive function by affecting the choroid plexus, which regulates the recruitment of immunoregulatory cells such as monocyte‐derived macrophages and Tregs into cerebral pathological sites (Baruch et al., 2015). A consistent finding is that IL‐10 (the main Tregs effector cytokine) signaling transduction is accelerated in the brains of AD patients, and IL‐10‐deficient APP/PS1 mice show restricted cerebral amyloidosis and less cognitive decline than controls via a rebalancing of abnormal innate immunity (Guillot‐Sestier et al., 2015). This result suggests that aberrant elevated IL‐10 signaling in AD patients may be a therapeutic target for AD.

In summary, T cells display abnormal phenotypes and dysfunction in AD, and transplantation or deletion of different T‐cell subtypes into AD mice has the potential to alter the progression of AD pathology. Accordingly, AD treatment strategies targeting T cells have been proposed (Table 3). However, it is still unclear whether these changes in T cells are related to disease progression. Thus, the critical question is whether abnormal T‐cell parameters are driving AD progression or do the abnormalities, including the lack of different phenotypes, occur only after onset of AD. Future work is needed to clarify the link between T‐cell abnormalities and disease severity. In particular, analyses of these abnormalities at different AD stages would be beneficial. These results should provide immunology‐based guidance for treating AD.

**TABLE 3:** Summary of AD therapy targeting T cells in animal models (studies in chronological order)

Study	Mouse model	Findings
(St‐Amour et al., 2014)	3xTg‐AD mouse	Intravenous immunoglobulin (IVIg) ameliorates cognitive performance and effector T cells are potential pharmacological targets of IVIg in AD model.
(Baruch et al., 2015)	5xFAD mouse	Transient depletion of Foxp3 (+) regulatory T cells (Tregs), or pharmacological inhibition of their activity, mitigates Alzheimer's disease pathology
(Guillot‐Sestier et al., 2015)	APP/PS1 mouse	Loss of IL−10, a Treg key cytokine, mitigates synaptic and cognitive deficits
(Marsh, Samuel E., et al. 2016)	Rag−5xFAD mouse	Loss of T, B, and natural killer (NK) cells appears to accelerate AD progression
(Baek et al., 2016)	3xTg‐AD mouse	Systemic transplantation of purified Tregs into 3xTg‐AD mice improved cognitive function and reduced deposition of Aβ plaques.
(Alves, Sandro et al. 2017)	APP/PS1 mouse	IL−2 administration induces systemic and brain regulatory T cells expansion and improves amyloid pathology, synaptic failure and memory.
(Laurent et al., 2017)	THY‐Tau22 mouse	Anti‐CD3 treatment prevented hippocampal T‐cell infiltration and reverted spatial memory deficit
(Yang, et al. 2020)	APP/PS1 mouse	Inactivated influenza vaccine (IIV) ameliorates cognitive deficits in APP/PS1 mice by breaking Treg‐mediated systemic immune tolerance.
(Brigas, Helena C et al. 2021)	3xTg‐AD mouse	γδ T cells are the major source of IL−17 in the CNS of 3xTg‐AD mice and neutralization of IL−17 prevents cognitive impairments and synaptic dysfunction

## 3 ASSOCIATION BETWEEN T CELLS AND THE CRITICAL FACTORS INVOLVED IN AD

### 3.1 ApoE modulates T‐cell activation

ApoE is a polymorphic protein involved in lipoprotein conversion and metabolism, produced by organs such as the brain, liver, kidneys, and spleen (Huang & Mahley, 2014). ApoE4, one of the protein isoforms of ApoE, interacts with Aβ more efficiently than ApoE3, which results in increased Aβ deposition and amyloid plaques in AD (Sanan et al., 1994; Schmechel et al., 1993; Strittmatter et al., 1993). ApoE4 can also elicit tau aggregation via the inhibition of noradrenaline transport (Kang et al., 2021). Therefore, AD risk is highly correlated with ApoE alleles (ε4>ε3>ε2), and ApoE4 is the biggest genetic risk factor for sporadic AD (Mahley & Rall, 2000; Theendakara et al., 2018). Besides, ApoE4 not only affects secreted ectodomain APPα (sAPPα) secretion (Cedazo‐Minguez et al., 2001; Vincent & Smith, 2001) but also directly regulates Sirtuin1 protein expression and enzyme activity (Theendakara et al., 2016), which is related to programmed cell death, insulin resistance, and synaptic function, all of which are involved in AD pathogenesis (Theendakara et al., 2018). Recent evidence revealed that ApoE4 can lead to BBB dysfunction in cognitively unimpaired individuals and yet more severe dysfunction in cognitively impaired individuals, independently of CSF Aβ and tau status (Montagne et al., 2020).

It has been shown that plasma lipoproteins containing ApoE have a role in inhibiting T‐cell activation and proliferation induced by PHA in vitro in an ApoE‐concentration‐dependent manner by downregulating the secretion of bioactive IL‐2 (Kelly et al., 1994; Macy et al., 1983). Consistent findings suggest that lack of ApoE exacerbates the production of proinflammatory factors including TNF‐α, IFN‐γ, IL‐12, and IL‐6 during LPS‐induced responses, whereas treatment with exogenous ApoE can normalize these cytokines levels (Ali et al., 2005). Similarly, in another neuroinflammation mouse model induced by ApoE deletion, an ApoE mimetic peptide reversed upregulated expression of proinflammatory factors (IL‐17, IL‐12, TNF‐α, IFN‐γ, IL‐6, and IL‐1β) (Wei et al., 2013). Moreover, two major proinflammatory T‐cell subtypes, Th1 and Th17 cells, were elevated in this model, and IL‐17 levels secreted by Th17 cells were elevated, promoting mononuclear cell infiltration and activation (Gao et al., 2010). Consistent with these results, treatment with IL‐17 antibody led to a significant amelioration of atherosclerotic symptoms (Smith et al., 2010). ApoE also disturbs the balance of Th17 and Treg in the spleen during arteriosclerosis; however, the mechanism remains unknown (Xie et al., 2010). The balance between Th17 and Treg is vital for regulating systemic inflammation and autoimmune response. Th17 cells promote immune system activation and pathogen clearance, while Treg cells protect organisms from damage by attenuating excessive inflammatory response. Therefore, ApoE is extremely important in the maintenance of immune homeostasis. These findings suggest that ApoE exerts an essential effect on the mediation of T‐cell function even though T cells may not be necessary in the formation of early‐early lesions. T cells most likely promote atherosclerosis by modulating monocyte recruitment, proliferation, and cytokine secretion in the later stages of AD.

Notably, humans expressing the ApoE4 isoform show a higher number of activated T cells than those expressing ApoE3 or ApoE2 (Bonacina et al., 2018). Consistently, AD patients with the ApoE allele 4 express higher Fas in T cells than those with the ApoE allele 3 (Lombardi et al., 2004), suggesting that different ApoE alleles are selective for T‐cell activation. Since cholesterol homeostasis in monocyte‐derived macrophages is dependent on ApoE isoform (Cullen et al., 1998), and cholesterol homeostasis has been identified as critical for regulating immune cells, including T cells (Ito et al., 2016; W. Yang et al., 2016; York et al., 2015), we hypothesize that ApoE may affect T‐cell function by promoting cholesterol and lipid metabolism in T cells. Given that ApoE is a crucial risk factor for AD and a significant modulator of T cells, we propose that ApoE may be an immunotherapy target for regulating abnormal T‐cell activation in AD.

### 3.2 Aβ regulates T‐cell activation

Amyloid precursor protein (APP) is sequentially cleaved by β‐secretase and γ‐secretase in an amyloidogenic pathway to produce Aβ, which aggregates into amyloid plaques (Cole & Vassar, 2007; Selkoe, 2001; Vassar, 2004). It is clear that the level of Aβ in the brain is significantly elevated during the progression of AD, which ultimately results in neuronal death and inflammation. Autoantibodies to Aβ have been detected and were found to be elevated in both AD patients and elderly AD mice, which suggests that Aβ can act as a self‐antigen to initiate humoral immune response (Nath et al., 2003). Moreover, Aβ‐reactive T cells were also detected in the peripheral blood of AD patients (Monsonego et al., 2013). The presentation of peripheral Aβ to T cells is typically detected in lymph glands; however, it has been reported that antigen‐presenting cells (APCs) can present Aβ to T cells infiltrating the parenchyma, although it is unknown why T cells recognize self‐antigen Aβ (Archambault et al., 2005).

The role of Aβ‐reactive T cells is complicated and controversial. On the one hand, it has been shown that Aβ‐reactive T cells in certain AD mouse models are beneficial for Aβ clearance via enhancement of microglial activation in an IFN‐γ‐dependent manner (Fisher et al., 2010; Monsonego et al., 2006). On the other hand, Aβ‐reactive T cells may be detrimental because they promote pathogenic immune responses in AD. These abnormal T cells lead to strong secretion of proinflammatory factors, including TNF‐α, IL‐1β, and IL‐6, contributing to chronic neuroinflammation and neurotoxicity (Mietelska‐Porowska & Wojda, 2017). Although the beneficial and detrimental effects of Aβ‐reactive T cells in Aβ pathology are still not fully elucidated, it has well accepted that Aβ as a specific antigen can be captured by APCs and then be recognized by T cells to induce T‐cell activation and proliferation. These processes are tightly related to AD pathology.

In addition to being presented as a specific antigen to T cells, Aβ may have a more direct effect on the regulation of T‐cell function; evidence had revealed that T cells can synthesize and secret APP upon activation to initiate the immune response (Bullido et al., 1996; Monning et al., 1990, 1992). Consistently, the lymphoblastoid cell line established from familial Alzheimer's disease (FAD) expresses higher APP than control patients (Matsumoto & Fujiwara, 1991). Therefore, T cells from FAD may express a higher level of APP. Furthermore, with the characteristics of a cell adhesion molecule, APP can bind to extracellular matrix components including collagen and laminin (Sondag & Combs, 2006), implying a role for APP in cell adhesion, cell–extracellular matrix contact, or cell–cell contact during T‐cell recruitment and infiltration.

Strikingly, data obtained in vitro have shown that synthetic APP peptides stimulate the proliferation of resting lymphocytes from young and old healthy donors, which correlates with IL‐2 expression (Trieb et al., 1996). It is a consistent finding that Aβ stimulation in vitro also significantly enhances T‐cell proliferation derived from AD patients and healthy elderly individuals (Jozwik et al., 2012). Moreover, exposure of Aβ to human activated T cells in vitro leads to increased secretion of proinflammatory factors, including IL‐6 and TNF‐α. However, administration of Aβ in vivo attenuates inflammation and reverses paralysis in a Th1/Th17‐induced experimental autoimmune encephalomyelitis (EAE) mouse model (Grant et al., 2012). Notably, both Aβ40 and Aβ42 directly inhibit CD4+ T‐cell activation and proliferation under aCD3/aCD28 antibody stimulation, and Aβ42 exhibits higher inhibitory properties. Moreover, APP expression in other leukocytes, such as monocytes, induces CCR5 expression and proinflammatory cytokine release (Giri et al., 2005; Sondag & Combs, 2006; Vehmas et al., 2004), which could, in part, modulate T‐cell activation.

Although the mechanisms whereby Aβ and APP regulate cellular T‐cell processes have not been fully elucidated, previous studies have suggested that Aβ can regulate T‐cell function in at least three distinct ways: (1) Aβ can be presented by APCs to T cells as an antigen and promote T‐cell activation, (2) Aβ precursor protein endogenously expressed in T cells or exogenous Aβ directly modulate T‐cell function, and (3) Aβ precursor protein expressed in monocytes induces proinflammatory cytokines which mediate T‐cell function indirectly. However, the exact mechanism that Aβ regulates the function of T cells may differ between different disease models and may be associated with the severity of the disease. It is reasonable to suggest that strategies which target Aβ for AD treatment may disturb the functional regulation of T cells by Aβ.

### 3.3 Alpha secretases regulate T‐cell function

Alpha secretases (α‐secretases) are members of the ADAM (a disintegrin and metalloproteinase) family, which cleave within the Aβ peptide to produce sAPPα and C83 in the non‐amyloidogenic pathway (Zhang et al., 2011). sAPPα, but not secreted ectodomain APPβ (sAPPβ), protects neurons against Aβ‐induced cytotoxicity and is thought to be a neurotrophic and neuroprotective factor (Tackenberg & Nitsch, 2019). Therefore, α‐secretases can facilitate AD prevention, not only by competitive cleavage of APP to preclude the formation of Aβ peptide but also by providing neuroprotective agents. Additionally, decreased activity of α‐secretase was observed in AD patients (Colciaghi et al., 2002; Kim et al., 2009). Therefore, pharmacological intervention targeting α‐secretase may provide a potential therapy for AD.

ADAM9, ADAM10, and ADAM17 (tumor necrosis factor‐α‐converting enzyme, TACE) have been identified as having α‐secretase activity (Buxbaum et al., 1998; Lammich et al., 1999). It has recently been shown that ADAM9 drives Th17‐cell development by producing bioactive transforming growth factor β1 (TGFβ1), and that T cells lacking ADAM fail to induce Th17‐dependent experimental autoimmune encephalomyelitis (Umeda et al., 2021). Besides, ADAM10 and ADAM17 were shown to regulate T‐cell function via the cleavage of lymphocyte activation gene 3 (LAG3), which must be cleaved to allow normal T‐cell activation (Li et al., 2007). ADAM10 and ADAM17 were also identified as major sheddases of T‐cell immunoglobulin and mucin domain 3 (Tim‐3), whereas Tim‐3 dampened the T‐cell response by inducing cell death (Moller‐Hackbarth et al., 2013). Thus, impaired activity of α‐secretases in AD may contribute to T‐cell abnormalities.

Levels of ADAM9 are significantly elevated in T cells from patients with systemic lupus erythematosus, and thus, T‐cell activity may regulate ADAM9 expression (Umeda et al., 2021). Indeed, further analysis revealed that the transcription factor inducible cAMP early repressor (ICER) can directly bind to *ADAM9* promoter and to promote ADAM9 transcription during T‐cell activation (Umeda et al., 2021). Moreover, TCR signaling transduction can also induce enzymatic activity of ADAM17 in a PKCθ‐dependent manner (Li et al., 2007). Given that ADAM exhibits neuroprotective properties in AD, T‐cell‐induced modulation of ADAM activity could be an alternative target for AD treatment.

### 3.4 T‐cell function is associated with β‐secretase

Beta‐secretase, known as β‐site amyloid precursor protein cleaving enzyme 1 (BACE1), cleaves the extracellular domain of APP to produce Aβ. The concentration and enzymatic activity of BACE1 in the CSF and blood of AD patients are significantly higher than that of control participants, indicating that BACE1 is a promising candidate biological marker of AD (Ewers et al., 2011; Hampel & Shen, 2009; Shen et al., 2018). Based on the amyloid hypothesis, Aβ is considered one of the leading potential causes of AD, so inhibition of BACE1 to reduce the production of Aβ is considered to be an effective strategy for AD treatment (Yan & Vassar, 2014). Besides APP, BACE1 has other substrates, including low‐density lipoprotein receptor‐related protein (LRP) (von Arnim et al., 2005), the voltage‐gated sodium channel (Nav1) β2 subunit (Navβ2) (von Arnim et al., 2005), and neuregulin‐1 (NRG1) (X. Hu et al., 2006; Willem et al., 2006), most of which are related to neuron and nervous system function. Furthermore, a recent study showed a BACE1‐dependent insulin receptor (IR) reduction in the liver of AD patients, which concluded that IRs are an additional novel substrate for BACE1, highlighting the role of BACE1 in type II diabetes and other metabolic disorders which are associated with underlying chronic insulin resistance (Meakin et al., 2018).

In addition to the nervous system and hepatic metabolism, there are also immune‐system‐related substrates of BACE1. For example, it has been shown that P‐selectin glycoprotein ligand‐1 (PSLG‐1) is a substrate for BACE1 and the cleavage site has been identified using mass spectrometry (Lichtenthaler et al., 2003). It is expressed in endothelial cells and leukocytes, including T cells, and binds to L‐selectin, E‐selectin, and P‐selectin to mediate monocyte adhesion during inflammation (da Costa Martins et al., 2007; Moore, 1998). In addition, there are another two inflammatory proteins, interleukin‐1 receptor 2 (IL‐1R2) and β‐galactoside α2, 6‐sialyltransferase (ST6Gal1), that are cleaved by BACE1 (Kuhn et al., 2007; Sugimoto et al., 2007). Correspondingly, BACE1 expression is tightly regulated by immune regulators, including nuclear factor kappa B (NF‐κB) (Cai et al., 2011), specificity protein 1 (Sp1) (Christensen et al., 2004), nuclear factor of activated T cells (NFAT) (Cho et al., 2008), GATA Binding Protein 1 (GATA‐1) (Lange‐Dohna et al., 2003), signal transducer and activator of transcription3 (STAT3) (L. Liu et al.,^2013^) and STAT1(Cho et al., 2007), all of which are key T‐cell transcription factors. More importantly, IFN‐γ, one of the proinflammatory factors secreted predominantly by T cells, has been reported to stimulate BACE1 expression and sAPPβ production (Hong et al., 2003). Surprisingly, recent studies identified that CD4+ T cells highly expressed BACE1, and BACE1 mediated T‐cell activation in the EAE and AD mouse model (Dai et al., 2021; Hernandez‐Mir et al., 2019).

In conclusion, these findings implicate an unexpected relationship between BACE1 and T cells. This relevance is bilateral because, on the one hand, changes of T‐cell‐related activity regulate BACE1 expression and activity. On the other hand, BACE1 may modulate T‐cell function via cleavage of various substrates expressed on leukocytes, including T cells.

### 3.5 The Role of γ‐secretase in T‐cell function

Gamma‐secretase, another crucial enzyme for the cleavage of APP into Aβ, is a membrane protein complex composed of four individual proteins: presenilin enhancer 2 (PEN2), presenilins (PS), nicastrin, and anterior pharynx defective 1 (APH‐1) (De Strooper, 2003; Kimberly et al., 2003). PSEN1 and PSEN2 are two homologous genes, respectively, encoding PS1 and PS2, representing the major catalytic subunits of γ‐secretase (Edbauer et al., 2003; Wolfe et al., 1999). PS1 and PS2 mutations are reportedly responsible for early‐onset familial Alzheimer's disease (EOFAD) (Haass & De Strooper, 1999; Sherrington et al., 1995; Sorbi et al., 1995). More than 70 known type I integral membrane proteins can be cleaved by γ‐secretase within their transmembrane domains (Wolfe, 2019). Among them, the best‐studied substrate of γ‐secretase is APP due to its relevance in AD (De Strooper et al., 1998). APP is consecutively cleaved by β‐secretase and γ‐secretase, which finally yield Aβ40 or Aβ42, the secreted ectodomain APPβ (sAPPβ) and APP intracellular domain (AICD) (Y. W. Zhang et al., 2011). Moreover, AICD displays transcriptional regulatory function by binding to target genes, including the tumor suppressor TP53, the Aβ‐degrading enzyme neprilysin (NEP), LRP1, and the epidermal growth factor receptor (EGFR) (Multhaup et al., 2015).

In addition to APP, the most studied substrate of γ‐secretase is the Notch receptor family (Shih Ie & Wang, 2007), which release the Notch intracellular domain (NICD) during the proteolysis for translocation to the nucleus and activation of transcription factors involved in cell development and cell fate determination (Henrique & Schweisguth, 2019; Kopan & Ilagan, 2009; Shih Ie & Wang, 2007). Over past decades, it was elucidated that the essential function of Notch signaling is mediated by γ‐secretase in the biological processes of T cells. Treatment with γ‐secretase inhibitors or deletion of Notch in hematopoietic progenitors and common lymphoid precursors impair the development of T cells (Hadland et al., 2001; Radtke et al., 1999; Wilson et al., 2001); conversely, retroviruses can induce continuous expression of Notch1 in hematolymphoid progenitors leading to thymic‐independent T‐cell development (Pui et al., 1999). Unsurprisingly, clinical trials of AD treatment with γ‐secretase inhibitors have failed due to their inhibition of Notch cleavage, resulting in abnormalities in the skin, gastrointestinal tract, thymus, and spleen (Imbimbo & Giardina, 2011). Notch‐signaling‐dependent regulation is necessary for the crucial proteins of T‐cell activation and differentiation, including NF‐κB, T‐bet, IFN‐γ, and TGF‐β, and this regulation can be abolished by γ‐secretase inhibitors (Amsen et al., 2015; Minter et al., 2005; Palaga et al., 2003; Samon et al., 2008; Shin et al., 2006). The Helbig and Maekawa research groups have shown that Notch signaling can prolong activated CD4+ T‐cell longevity and promote memory CD4+ T‐cell maintenance (Helbig et al., 2012; Maekawa et al., 2015). Furthermore, CD44, a critical regulator that controls T‐cell development and function (Baaten et al., 2012; Graham et al., 2007), has been identified as a novel substrate of γ‐secretase in vitro (Lammich et al., 2002), implying a novel mechanism of γ‐secretase in the regulation of T‐cell function.

In summary, the role of γ‐secretase in most biological T‐cell processes suggests that γ‐secretase inhibitors should be used with caution to avoid affecting normal physiological T‐cell function. Conversely, γ‐secretase inhibitor treatment may be an effective strategy for the rescue of abnormal T‐cell function under certain conditions.

### 3.6 T cells contribute to Tau pathology

Tau is a microtubule‐binding protein abundant in neurons and is mainly localized on axons and dendrites (Hirokawa et al., 1996; Ittner et al., 2010; Konzack et al., 2007; Utton et al., 2002). The primary role of tau in neurons is to modulate the stability of axonal microtubules by interacting with tubulin. While phosphorylation is a common post‐translational modification of tau (Cleveland et al., 1977; Ksiezak‐Reding et al., 2003; Sengupta et al., 1998), abnormal hyperphosphorylation of tau leads to NFT formation and is neurotoxic in neurodegenerative diseases, including AD (Kenessey et al., 1995; Kopke et al., 1993; Ksiezak‐Reding et al., 1992). Under pathological conditions, excessive or abnormal phosphorylation of tau which aggregates into NFTs is responsible for synaptic dysfunction and neuronal death (Guo et al., 2017).

Tau‐specific CD4+ T cells are widely distributed in the peripheral blood from the general population (Lindestam Arlehamn et al., 2019). These cells collected from either young or healthy elderly donors exhibited reactivity to tau‐ and p‐tau‐derived peptides associated with IL‐5 and IFN‐γ. The presence of the tau‐autoreactive T cells indicates that the thymic selection of CD4+ T cells is not sufficient to eliminate these cells. Furthermore, extravascular T cells are observed in the brains, specifically in the hippocampus, of AD patients, most of which are CD8+ T cells. These extravascular T cells are correlated with tau pathology but not with Aβ pathology (Merlini et al., 2018), suggesting that T cells could be critical for driving the tau‐dependent phase of the AD pathology.

Interestingly, T‐cell infiltration has been discovered positively correlated with p‐tau load in the inferior parietal lobule, middle temporal gyrus, and medial frontal gyrus of AD patients (Zotova et al., 2013). In addition, T‐cell infiltration was also observed in the cortex of frontotemporal dementia patients with P301L tau mutation and the hippocampus of THY‐Tau22 mouse model (Laurent et al., 2017). These data support an instrumental role of tau‐driven pathophysiology in brain T‐cell infiltration. However, the underlying molecular mechanisms are not clear.

All these findings together suggest that T cells can respond to, as well as contribute to, tau‐driven pathology. Given the high levels of tau aggregation in the elderly, T‐cell responses to tau may contribute to the progression of neurological diseases, including AD and PD. As tau‐driven pathology in AD induces synaptic loss, cytoskeletal dysfunction, and impair axonal transport, tau‐targeted therapeutic strategies have been proposed. Immunization against tau has shown great potential to treat tau pathology by inhibiting tau transmission and aggregation (Kfoury et al., 2012; Yoshiyama et al., 2013). Furthermore, it has been demonstrated that passive immunization may be more effective and safer than active immunization (Spillantini & Goedert, 2013). It is worth pointing out that it is not known whether tau‐targeted treatment is involved in the regulation of T‐cell response and whether this occurs without breaking down the functional immune homeostasis.

### 3.7 T‐cell activity is associated with neuronal loss

Brain atrophy caused by neuronal and synaptic loss is one of the definitive pathological lesions observed in AD. Several neuronal death mechanisms have been determined in AD (Cotman & Su, 1996; Niikura et al., 2006). Substantial evidence suggests that Aβ plays a significant role in initiating neurotoxicity and neuronal dysfunction and results in neuronal death. Accumulated Aβ‐initiated toxicities are characterized by mitochondrial dysfunction, oxidative stress, and calcium dyshomeostasis in neurons (Canevari et al., 2004; Caughey & Lansbury, 2003; LaFerla, 2002). Aβ also alters the acetylcholinesterase (AChE) activity, promotes the modification of c‐Fos with O‐linked β‐N‐acetyl glucosamine (O‐GlcNAc), and increases p‐tau expression in neurons, all of which contribute to neuronal apoptosis (Choi et al., 2019; Song et al., 2018). Moreover, Aβ oligomers trigger cyclin‐dependent kinase‐5 (Cdk5) activation, which thereafter mediates neuronal apoptosis via induction of p53 phosphorylation (Lapresa et al., 2019). In addition to the toxic effects of Aβ, inhibition of the proteasome is sufficient to induce neuronal apoptosis by an increase in poly‐ADP‐ribosylation, elevated activation of caspase‐3‐like proteases, and accelerated amylospheroid (Keller & Markesbery, 2000; Komura et al., 2019; Qiu et al., 2000). Widespread proinflammatory factors in the brain such as TNF‐α, IFN‐γ, and IL‐1β also have significant detrimental effects on neurons (Barker et al., 2001; Brown & Neher, 2010; Combs et al., 2001; Rothwell, 2003).

The migration of T cells into the CNS parenchyma during the pathological process of AD has attracted attention due to the crosstalk between neurons and T cells. Nitsch and colleagues were first to present the process of direct physical contact between neurons and T cells in living brain tissue (Nitsch et al., 2004). Notably, they showed that T‐cell–neuronal interactions induced calcium oscillations in neurons, resulting in neuronal death; however, a combination of glutamate receptor antagonists and perforin blockade were sufficient to inhibit this detrimental effect on neuronal calcium levels. Later, researchers pointed out that, although CD8+ T cells exerted more toxicity than CD4+ T cells in a neuronal co‐cultured system, CD4+ T cells can promote cytotoxicity of CD8+ T‐cell neurons via induction of violent inflammation (Zhao et al., 2018). This phenomenon may be due to the lack of MHC II molecule expression on neurons; thus, CD4+ T cells cannot directly interact with neurons in an antigen‐specific way, while neurons can constitutively express the MHC I molecule recognized by CD8+ T cells. Significantly, T cells can also contribute to neuronal loss by releasing proinflammatory cytokines. For instance, IL‐17 secreted by Th17 cells induces neuronal cell death by IL‐17—IL‐17R signaling and NF‐κB activation (Sommer et al., 2018). A consistent finding has been shown that Th17 cells infiltrating the brain can directly induce the death of dopaminergic neurons and promote the activation of glial cells via LFA‐1/ICAM‐1‐mediated intercellular communication involving c‐Jun NH(2)‐terminal kinase (JNK)/ activator protein 1 (AP‐1) signal activation in neurons (Liu et al., 2017).

It has been argued that T cells exhibit neuroprotective properties aside from neurotoxic effects during pathological situations (Baek et al., 2016; Chiu et al., 2008; Endo et al., 2015; Moalem et al., 1999). Most importantly, T cells are indispensable for spatial learning and the maintenance of neurogenesis under physiological situations in adulthood (Ziv et al., 2006). More work is required to discriminate the contributions from different T‐cell subtypes to neurons in neurodegenerative disorders and to determine their pathogenic and neuroprotective properties. Understanding the particular effects of T cells in different diseases may provide important information to be carefully considered when developing therapeutic strategies.

### 3.8 T cells contribute to neuroinflammation

Pathogenesis of AD is not limited to neuronal loss but also extends to extensive glial cell activation (Lee & Landreth, 2010; Medeiros & LaFerla, 2013; Molofsky et al., 2012; Morales et al., 2013; Paresce et al., 1996). In AD, neuroinflammation is a common phenomenon activated by amyloid plaques and NFTs. It contributes to pathogenesis just as much as plaques and tangles, perhaps even more so, and there is evidence to suggest that neuroinflammation is a critical modulator of AD development (Heneka et al., 2015; Jiang et al., 2014; R. Li et al., 2004; Van Eldik et al., 2016; L. Yang et al., 2002).

Microglia, the most abundant resident innate immune cells in the brain, are vital cellular mediators for initiating neuroinflammatory responses. Various immune‐related receptors are expressed on microglia cell membranes, including scavenger receptors, chemokine receptors, cytokine receptors, and pattern‐recognition receptors (PRRs), which can bind with proinflammatory mediators to trigger microglial activation (Kierdorf & Prinz, 2013). Furthermore, Genome‐Wide Association Studies (GWAS) have also determined many gene mutations related to an elevated risk of late‐onset AD (LOAD) and most are expressed abundantly in microglia, such as triggering receptor expressed on myeloid cells 2 protein (TREM2), complement receptor type 1 (CR1) and CD33 (Karch & Goate, 2015). Recently, high‐throughput sequencing methods at the single‐cell level (scRNA‐seq) have been applied to study microglial function and heterogeneity throughout microglial lifespan and AD (Hammond et al., 2019; Keren‐Shaul et al., 2017; Q. Li et al., 2019; Mathys et al., 2017; Zeisel et al., 2015). Mathys and colleagues first used the scRNA approach to track activation of microglia during the neurodegenerative processes in CK‐p25 mice (Mathys et al., 2017), which mimic the major pathological hallmarks of AD, such as impaired synaptic plasticity, upregulated Aβ, and neuronal death. They found that proinflammatory factors TNF‐α and macrophage migration inhibitory factor (MIF) were upregulated in the early microglial response state (1‐week post p25 induction), suggesting that inflammation occurs as early as Aβ production and may initiate cascading effects that ultimately lead to neuronal loss and cognitive dysfunction. Astrocytes are another key regulator of neuroinflammation. During neuroinflammation, destructive signaling pathways in astrocyte are triggered by IL‐17, sphingolipids, and LacCer, followed by activation of the NF‐κB‐dependent and STAT3‐dependent transcription of proinflammatory factors, which finally contributes to neuroinflammation and promotes neurodegenerative disorders.

It is clear that T cells infiltrate the CNS and promote neuroinflammation during the pathogenesis of AD (Hoppmann et al., 2015; Laurent et al., 2017; Mietelska‐Porowska & Wojda, 2017; Raveney et al., 2015; Wimmer et al., 2019; Yu et al., 2015). Th1 and Th17 cells significantly accumulate in the brains of APP/PS1 mice; however, only Aβ‐specific Th1 cells adoptively transplanted to APP/PS1 mice lead to a deficit in cognitive function (Browne et al., 2013). These Aβ‐specific Th1 cells promote microglia activation and neuroinflammation via IFN‐γ production, and administration of a neutralizing IFN‐γ antibody reverses the outcomes of Th1 cells on microglia activation and Aβ deposition. In addition, microglial cells co‐cultured with Aβ‐specific Th1 cells or Aβ‐specific Th17 cells induce proinflammatory cytokine production in microglia, which can be attenuated by Th2 cells (McQuillan et al., 2010), indicating that the regulation of microglia activation by T cells occurs in a cell‐type‐dependent manner. Surprisingly, T cells, especially brain‐resident CD4+ T cells, can regulate microglia activation and are required for microglia maturation (Pasciuto et al., 2020). The absence of CD4+ T cells traps microglia in a fetal‐like transcriptional state and results in defective synaptic pruning and depression‐like mouse behavior. Besides, T cells can positively contribute to astrocyte activation and then exacerbate neuroinflammation. T‐cell‐derived IFN‐γ induces astrocyte proliferative response in vitro and promotes brain reactive astrogliosis (Yong et al., 1991). Moreover, IL‐17, produced by Th17 cells, has been repeatedly identified as an effective astrocyte stimulator. IL‐17 stimulates inducible nitric oxide synthase activation (Trajkovic et al., 2001), regulates macrophage inflammatory proteins‐1α (MIP‐1α) expression via PI3K/Akt and NF‐κB pathways (Yi et al., 2014), and enhances the IL‐6 signaling pathway (Ma et al., 2010) in astrocytes. In addition to activating microglia and astrocytes, T cells may also promote brain inflammation by inducing myeloid cells, including dendritic cells (DCs) and macrophages associated with secretion of TNF‐α, IL1β, and IL‐6 (Town et al., 2005b). More critically, T cells not only contribute to neuroinflammation but also initiate neuroinflammation in an MHCII‐dependent manner. In this way, conventional DCs process and present myelin antigen to parenchymal T cells and then trigger T cells to infiltrate the CNS to initiate neuroinflammation (Mundt et al., 2019).

Of particular note, T cells also exhibit neuroprotective properties by regulating the trophic/cytotoxic glial cell balance and restoring glial cell activation (Beers et al., 2008). CD4+ T‐cell‐derived IL‐10 and IL‐4 are two immunoregulatory factors with important neuroprotective properties, involving the inhibition of microglia with subsequent reduction of nitric oxide and TNF‐α levels (Chao et al., 1993; Frenkel et al., 2005).

Summarizing, these results highlight T cells as an essential modulator in mediating neuroinflammation, which is achieved by activating microglia and astrocytes and releasing proinflammatory factors, implicating T cells as a potential immunotherapy target for neuroinflammation in neurodegenerative disease.

## 4 CONCLUDING REMARKS

In this article, we first reviewed the abnormal behavior of T cells in the progression of AD. Although the significance of T cells in AD pathogenesis is still hotly debated, there is convincing evidence from pre‐clinical, epidemiological, and genetic studies which indicate that the immune system is closely involved in AD and that T cells contribute to the pathological responses of AD (Mietelska‐Porowska & Wojda, 2017; Schellenberg, 2012; Wyss‐Coray & Rogers, 2012).

We then discussed in detail factors which affect T‐cell function in AD. We summarized that the ApoE might affect T‐cell activation via regulation of lipid metabolism. In contrast, the mechanisms by which Aβ regulates T‐cell function are multiplex, including antigen presentation, direct modulation of endogenous or exogenous Aβ, and indirect activation via monocytes. Besides, tau‐driven pathology also contributes to T‐cell activation, although the exact mechanism remains unknown. Moreover, α‐, β‐, and γ‐secretase, three crucial APP enzymes, modulate T‐cell function, in a manner which is dependent for the most part on the cleavage of specific substrates. As for neuronal loss, we should notice the pathogenic contribution of T cells and the neuroprotective properties of different T‐cell subsets. Lastly, neuroinflammation, one of the crucial hallmarks and contributors of AD, can facilitate the activation and recruitment of T cells into the brain. At the same time, activated T cells exacerbate AD pathology via the activation of microglia and astrocytes and through the release of proinflammatory cytokines.

**FIGURE 2:**
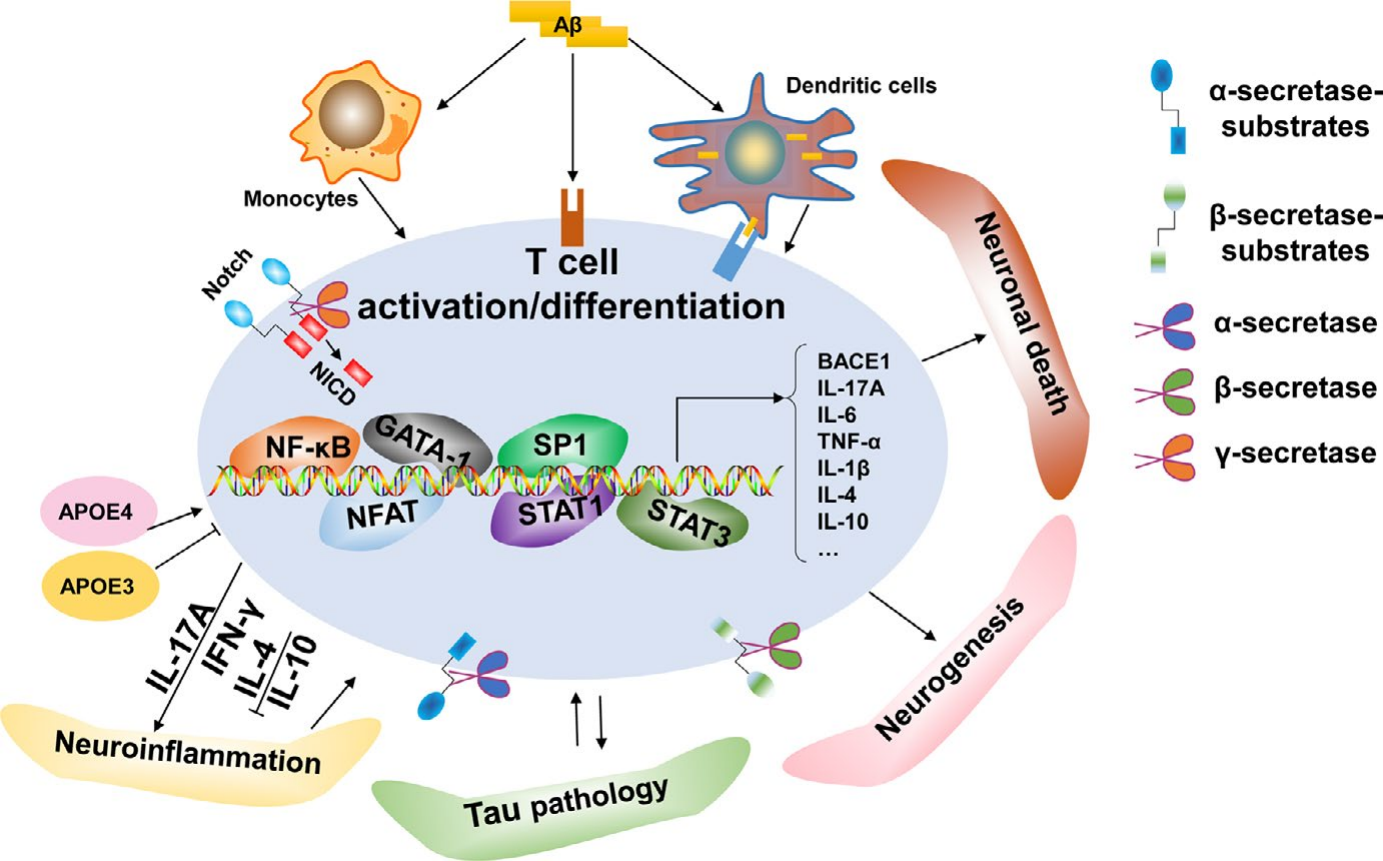
Schematic representation of the association between T cells and AD hallmarks. (1) ApoE is a key risk factor for AD and is also a significant T‐cell modulator, a function which may be ApoE‐allele‐dependent. (2) (a) Aβ can be presented by APCs to T cells as an antigen and promote T‐cell activation; (b) Aβ precursor protein endogenously expressed in T cells or exogenous Aβ directly modulates T‐cell function; (c) Aβ precursor protein expressed in monocytes induce proinflammatory cytokines to indirectly mediate T‐cell function. (3) α‐secretase mediates T‐cell function via the cleavage of diverse substrates, whereas T‐cell activation promotes α‐secretase activity. (4) T‐cell‐related biological changes regulate BACE1 expression and activity. Conversely, BACE1 may modulate T‐cell function via the cleavage of various substrates expressed on T cells. (5) The Notch receptor family are substrates of γ‐secretase, which releases the Notch intracellular domain (NICD) during proteolysis for translocation to the nucleus and activation of transcription factors involved in T‐cell development and T‐cell fate determination. (6) T cells are correlated with tau pathology and tau‐driven pathology may also induce excessive activation of T cells. (7) T cells migrate into the CNS parenchyma during the pathological progression of AD and contribute to neuronal death while T cells are also neuroprotective for spatial learning and the maintenance of neurogenesis under physiological situations. (8) T cells infiltrate the CNS and promote neuroinflammation during the pathogenesis of AD. Notably, T cells may also exhibit neuroprotective properties by regulating trophic/cytotoxic glia balance and restored glial activation

In conclusion, we summarized that AD risk factors and hallmarks can modulate T‐cell function, and abnormal activation of T cells in AD can also act on these critical factors, ultimately exacerbating AD pathology (Figure 2). Thus, understanding these causal associations may provide important insights into developing effective therapeutics. We also proposed that targeted treatment based on these risk factors and hallmarks may cause changes in the normal T‐cell phenotypes and peripheral immune responses under the physiological state. Therefore, the critical question is how to identify and limit the potential side effects of AD‐related factor‐based therapies on the normal T‐cell function.

## COMPETING INTERESTS

All authors declare that they have no competing financial interests.

## AUTHOR CONTRIBUTIONS

Linbin Dai and Yong Shen made substantial contributions to the review including writing the manuscript, preparing the figures and tables, and discussing the content.

## Data Availability

The data that support the findings of this study are available from the corresponding authors upon reasonable request.
